# Pathology of Bursae of Fabricius in Methionine-Deficient Broiler Chickens

**DOI:** 10.3390/nu5030877

**Published:** 2013-03-13

**Authors:** Bangyuan Wu, Hengmin Cui, Xi Peng, Jing Fang, Wei Cui, Xiaodong Liu

**Affiliations:** Key Laboratory of Animal Diseases and Environmental Hazards of Sichuan Province, College of Veterinary Medicine, Sichuan Agricultural University, Ya’an 625014, China; E-Mails: wubangyuan2008@163.com (B.W.); pengxi197313@163.com (X.P.); fangjing4109@163.com (J.F.); yonex_1981@163.com (W.C.); liuxiaodongk@yahoo.com.cn (X.L.)

**Keywords:** broilers, bursa of Fabricius, flow cytometry (FCM), methionine deficiency, pathology

## Abstract

The purpose of this 42-day study was to investigate the effects of methionine (Met) deficiency on immune function by determining the relative weight, morphological and ultrastructural changes of bursae of Fabricius, cell cycle, and apoptosis of bursa cells. One hundred and twenty one-day-old avian broilers were randomly divided into two groups and fed on a control diet (starter diet, Met 0.50%; grower diet, Met 0.40%) and Met-deficient diet (starter diet, Met 0.26%; grower diet, Met 0.28%) for six weeks. The relative weight of bursae was decreased with Met deficiency when compared to that of the control group. Lesions were also observed in the Met-deficient group. Histopathologically, the numbers of lymphocytes in the follicles were decreased. Ultrastructurally, the mitochondria of lymphocytes were swollen in the Met-deficient group. As measured by flow cytometry, bursal cells in the G_0_G_1_ phase were significantly higher (*P* < 0.01), and bursal cells in the S, G_2_M phases and proliferating index were obviously lower (*P* < 0.01) with Met deficiency than in the control group. Moreover, the percentage of apoptotic cells in the bursae were significantly increased in Met-deficient birds (*P* < 0.01). It was concluded that Met deficiency restrained the development of the bursae of Fabricius and affected the humoral immunity of the chickens.

## 1. Introduction

Methionine (Met) is the first limiting essential amino acid and can improve growth performance and carcass quality of poultry [1,2,3]. Met deficiency could depress body weight gain, food intake and efficiency of food utilization in chicks [4]. As a sulfur amino acid intermediate in the methylation and transsulfuration pathways [5], Met is the precursor of homocysteine and is the initiating amino acid in the synthesis of eukaryotic proteins. Met metabolism plays a crucial role in the cellular assimilation of folate [6]. One essential function of Met in neurulation may be as precursor for *S*-adenosylmethionine (SAM) and the methyl donor in transmethylation reactions [7], which plays a main role as the biological methyl donor for the methylation of DNA, RNA, and protein [8]. Moreover, Met is a protective factor against various types of liver damage. For example, it has been shown to prevent lipid accumulation in the liver [9,10].

Apart from these functions, Met is also involved in avian immune functions. Dietary Met can promote antibody production and cell-mediated immune responses in broilers [11,12,13]. Met is one of the components of the antibody response, which might be related to T-cell help [12]. Met is also required for production of interleukin-1 in immunologically challenged chicks [14]. It is reported that antibody production to sheep erythrocytes (SRBC) is decreased and splenocyte proliferative responses to concanavalin A (ConA) are also decreased in Met deficiency, which can preferentially affect cell-mediated immune responses relative to the development of the lymphoid organs and antibody production in chickens [15]. Also, Met deficiency can result in decreased humoral and nonspecific immunocompetence (serum lysozyme activity and phagocytosis of neutral red of peripheral blood lymphocytes) of broilers [16]. It is also reported that the cells (both proliferating PC12 cells and postmitotic neurons isolated from fetal rat brains) undergo apoptosis due to deprivation of other individual essential nutrients (such as Met) [17]. 

The bursa of Fabricius is the primary lymphoid organ, and is responsible for the establishment and maintenance of the B-cell compartment in avian species [18,19]. According to the aforementioned references, there have been few systematic reports thus far on the effect of Met deficiency on the bursae of Fabricius of broiler chickens. In the present research, the experiment was conducted with the objective of examining the effects of Met deficiency on the bursa, including lesions, the cell cycle, and apoptosis of bursae, before evaluating the immune function of chickens by methods of experimental pathology, flow cytometry (FCM) and immunohistological methods, in order to provide helpful materials for similar studies in both human and other animals in the future.

## 2. Materials and Methods

### 2.1. Chickens and Diets

One hundred and twenty one-day-old healthy avian broilers (obtained from Wenjiang poultry farm, a commercial rearing farm in Sichuan province) were randomly allotted by body weight to two separate feed treatment groups of 60 broilers each (6 replicates of 10 birds per treatment group). Broilers were fed either a control diet or a Met deficient diet. Birds were housed in cages with electrical heaters and were provided feed and water *ad libitum* for 42 days.

Experimental diets were formulated by NRC (1994) [20]. The Met content of the Met-deficient diet was measured by HITACHI L-8800 automatic amino acid analyzer. The results showed that the Met content in the starter diet was 0.26%, and Met content in the grower diet was 0.28%. Met, 0.24% and 0.12%, respectively, were added to the Met-deficient diets to produce the control diets (starter diet, Met 0.50%; grower diet, Met 0.40%).

At day 7, 14, 21, 28, 35, and at 42 days of age, five broilers in each group were sacrificed for observation and determination. 

### 2.2. Clinical Signs and Relative Bursa Weights

Clinical signs were observed daily. At each collection date, five birds randomly selected from each treatment group were euthanized and necropsied. Microscopic changes of bursae were observed and recorded. Bursae were dissected from each broiler and weighed after dissecting connective tissue around the organ. Related weight (RW) of bursae was calculated by the following formula:

RW = organ weight/body weight (g/kg)

### 2.3. Pathological Observation

After obtaining weight, bursae were fixed in 4% buffered formaldehyde and routinely processed in paraffin.

The method: Thin sections (5 μm) of each tissue were sliced from each block and mounted on glass. Slides were stained with hematoxylin and eosin (H and E). Histological slides were examined under an Olympus light microscope (Olympus, Japan). 

At the end of the experiment (42 days of age), three broilers in each group were euthanized and then immediately necropsied as described by Peng *et al*. [21]. Bursae were dissected and then were fixed in 0.5% glutaraldehyde and postfixed in 2% Veronal acetate-buffered OsO_4_. After dehydration in graded alcohol, it was embedded in Araldite. The blocks were sectioned in a microtome with a glass knife. The sections, 65–75-nm thick, were placed in uncoated grids. The sections were stained with uranyl acetate and poststained with 0.2% lead citrate and examined with an H-600 electron microscope. 

### 2.4. Cell Cycle of Bursa of Fabricius

At 14, 28, and 42 days of age, broilers in each group were selected for the determination of bursa of Fabricius cell cycle stages in the bursa of Fabricius by flow cytometry, as described by Cui *et al.* [22].

The method: Five broilers in each group were humanely killed at 14, 28, and 42 days of age, and thymus were immediately taken from each chicken and ground to form a cell suspension that was filtered through a 300-mesh nylon screen. The cells were washed twice with cold PBS (phosphate buffer solution, pH 7.2–7.4), and suspended in 1× binding buffer (Cat. No. 51-66121E) at a concentration of 1 × 10^6^ cells/mL. Five hundred microliters of the solution was transferred to a 5 mL culture tube, cells were briefly centrifuge (500–1000 rpm), and the supernatant was decanted. Five microliters 0.25% Tritonx-100 and 5 μL PI (Cat. No. 51-66211E) was added. Cells were gently vortexed and incubated for 30 min at RT (25 °C) in the dark. Finally, 500 μL of PBS was added to each tube and cells were analyzed by flow cytometry (BD FACSCalibur) within 45 min of preparation. The results were analyzed by Mod Fit LT for Mac V3.0 program.

### 2.5. Annexin-V Apoptosis Detection by Flow Cytometry

Simultaneously, bursae were sampled from each broiler for the determination of the percentage of apoptotic cells by flow cytometry, as described by Peng *et al*. [21].

The method: Five broilers in each group were humanely killed at 14, 28, and 42 days of age, and spleens were immediately taken from each broiler and ground to form a cell suspension that was filtered through a 300-mesh nylon screen. The cells were washed twice with cold PBS (phosphate buffer solution, pH 7.2–7.4) and were then suspended in 1× binding buffer (Cat. No. 51-66121E) at a concentration of 1 × 10^6 ^cells/mL. One hundred microliters of the cell suspension were transferred to 5 mL culture tubes, and 5 μL of Annexin V-FITC (Cat. No. 51-65874X) and 5 μL of PI (Cat. No. 51-66211E) were added. The mixture was gently vortexed and incubated for 15 min at 25 °C in the dark. Four hundred microliters of 1× binding buffer was added to each tube, and analyzed by flow cytometry (BD FACSCalibur) within 1 h of cell preparation. The results were analyzed by Cell Quest software.

### 2.6. Statistical Analysis

Data were subjected to Independent-Samples *T* test using SPSS 11.0 software and presented as means ± standard deviation. Differences between means were assessed, and values of *P* < 0.05 were considered significant.

## 3. Results

### 3.1. Clinical Observation

Broilers with Met deficiency grew slower (Table 1) and showed slightly decreased feed intake. In addition, no clinical signs of illness were observed.

**Table 1:** Effect of Met deficiency on the body weight (g) of broilers.

Groups	7 days	14 days	21 days	28 days	35 days	42 days
Control group	148.40 ± 3.05	354.27 ± 9.24	657.73 ± 20.11	999.80 ± 28.16	1347.40 ± 54.42	1781.33 ± 78.18
Met Deficiency	138.40 ± 2.38	313.73 ± 5.52 **	543.60 ± 10.69 **	808.20 ± 16.60 **	1182.00 ± 28.93 *	1562.93 ± 37.13 *

Data are the means ± standard deviation (*n* = 5); * *P* < 0.05, compared with the control group, ** *P* < 0.01, compared with the control group; Data were analyzed by Independent-Samples *T* test using SPSS 11.0 software.

### 3.2. Changes of Weight and Relative Weight of Bursa

No significant differences were noted between Met deficiency and control group at 7 and 14 days of age. At 21 days of age, the weight and relative weight of bursae were significantly lower in Met deficiency than the control group (*P* < 0.05 or *P* < 0.01). At 28, 35, and 42 days of age, the weight and relative weight of bursae were significantly decreased in Met deficiency, when compared with those of control group (*P* < 0.01). The results were showed in Table 2.

**Table 2:** Effect of Met deficiency on weight and relative weight of the bursa of Fabricius.

Item	Groups	7 days	14 days	21 days	28 days	35 days	42 days
Weight of bursae (g)	Control group	0.19 ± 0.02	0.58 ± 0.05	1.41 ± 0.16	1.88 ± 0.14	2.84 ± 0.42	3.90 ± 0.46
	Met deficiency	019 ± 0.02	0.53 ± 0.05	1.05 ± 0.11 *	1.12 ± 0.24 **	1.29 ± 0.28 **	2.17 ± 0.43 **
Relative weight of bursae (g/kg)	Control group	1.57 ± 0.01	1.69 ± 0.01	2.08 ± 0.01	1.79 ± 0.01	1.87 ± 0.01	1.93 ± 0.01
	Met deficiency	1.50 ± 0.02	1.56 ± 0.01	1.69 ± 0.01 **	1.40 ± 0.01 **	1.53 ± 0.01 **	1.38 ± 0.01 **

Data are the means ± standard deviation (*n* = 5); * *P* < 0.05, compared with the control group, ** *P* < 0.01, compared with the control group; Data were analyzed by Independent-Samples *T* test using SPSS 11.0 software.

### 3.3. Pathological Lesions of Bursae of Fabricius

**Figure 1:**
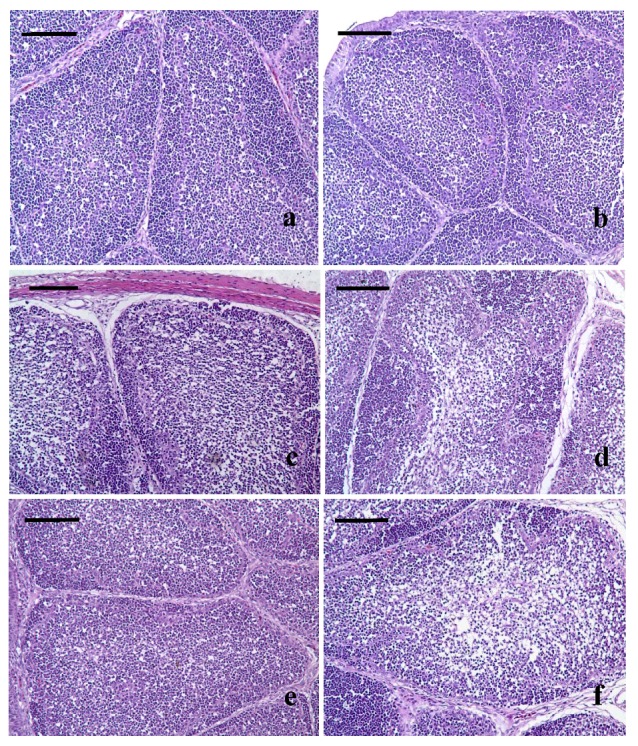
(**a**) At 21 days of age, lymphocytes are slightly reduced in lymphoid follicles with thinner cortices and wider medullae observed in the Met-deficient birds (**b**) when compared with those of the control group (**a**). (**c**) At 28 days of age, the bursal lymphoid follicles of birds from the Met-deficient group had decreased lymphocyte numbers decreased with thinner cortices and wider medullae. (**d**) At 35 days of age, the lymphocytes are significantly decreased in the Met-deficient birds’ lymphoid follicles. (**e**) The lymphoid follicles at 42 days of age in the control group. (**f**) At 42 days of age, the lymphocytes are significantly decreased in lymphoid follicles with a thinner cortex and wider medulla in Met-deficient birds. H and E Bars = 50 µm.

Lesions were histopathologically observed in Met deficiency from 21 days of age to the end of the experiment. At 21 days of age, the lymphocytes of bursae were slightly decreased in lymphoid follicles with a thinner cortex and wider medulla in Met deficiency (Figure 1b) when compared with those of the control group (Figure 1a). From 28 to 42 days of age, hypocellular became gradually more pronounced. At 28 days, the lymphocytes were decreased in lymphoid follicles with thinner cortices and wider medullae being observed in the bursae of the Met-deficient treatment group (Figure 1c). At 35 and 42 days of age, the lymphocytes were significantly decreased in lymphoid follicles with a thinner cortex and wider medulla in the bursa with Met deficiency (Figure 1d–f).

### 3.4. Ultrastructural Changes

**Figure 2:**
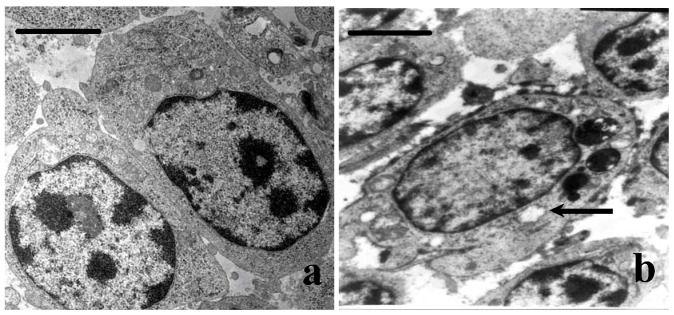
The ultrastructural changes of bursae at 42 days of age. (**a**) The lymphocytes in the control group. (**b**) The mitochondria of lymphocytes are swollen or vacuolated with degenerating cristae (*right arrow*) in Met-deficient birds (Bar = 2 μm).

No ultrastructural changes were observed in the control group (Figure 2a). The mitochondria of lymphocytes were swollen and vacuolated with degenerating cristae in Met-deficient birds (Figure 2b). 

### 3.5. Cell Cycle Phase Distribution of Bursa Cells

To examine the dynamics of cell cycle progression, G_0_G_1_, S and G_2_M phase distribution of the bursal cells in the Met-deficient group were observed when compared with those of the control group. G_0_G_1_ phase cell distribution gradually accumulated from 14 to 42 days of age and became significantlyincreased (*P* < 0.01) with Met deficiency. However, G_2_M, S phase cell distribution, and proliferation index (PI) were decreased in the bursal cells of Met-deficient birds of 14 and 42 days of age (*P* < 0.01). The results are shown in Table 3.

**Table 3:** Effect of Met deficiency on cell cycle phase distribution of bursae of Fabricius.

Groups	Item	14 days	28 days	42 days
Control group	G_0_G_1_	82.93 ± 0.36	83.84 ± 0.57	87.95 ± 0.31
	S	8.55 ± 0.05	7.97 ± 0.57	5.83 ± 0.23
	G_2_M	8.65 ± 0.24	8.18 ± 0.67	6.22 ± 0.08
	PI ^a^	17.14 ± 0.39	16.16 ± 0.56	11.93 ± 0.35
Met deficiency	G_0_G_1_	85.91 ± 0.42 **	88.24 ± 0.21 **	93.66 ± 0.40 **
	S	6.96 ± 0.31 **	5.44 ± 0.11 **	2.84 ± 0.06 **
	G_2_M	7.33 ± 0.15 **	6.32 ± 0.21 **	3.38 ± 0.44 **
	PI ^a^	14.09 ± 0.43 **	11.76 ± 0.21 **	6.22 ± 0.40 **

^a^ PI = 
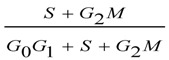
 × 100%; Data are the means ± standard deviation (*n* = 5); * *P* < 0.05, compared with the control group, ** *P* < 0.01, compared with the control group; Data were analyzed by Independent-Samples *T* test using SPSS 11.0 software.

### 3.6. Annexin-V-FITC Staining Assay by Flow Cytometry

Annexin V-FITC was used to quantitatively determine the percentage of cells that were actively undergoing apoptosis. The percentage of apoptotic cells in the bursae were significantly increased in Met deficient birds at 28 days and 42 days when compared with that of the control group (*P* < 0.05 or *P* < 0.01). The results were showed in Table 4. 

**Table 4:** Effect of Met deficiency on the percentage of apoptotic cells (%) in the bursa of Fabricius.

Group	14 days	28 days	42 days
Control group	1.99 ± 0.03	2.48 ± 0.29	3.19 ± 0.34
Met deficiency	2.13 ± 0.10	3.20 ± 0.27 *	5.74 ± 0.54 **

Data are the Means ± standard deviation (*n* = 5); * *P* < 0.05, compared with the control group, ** *P* < 0.01, compared with the control group; Data were analyzed by Independent-Samples *T* test using SPSS 11.0 software.

## 4. Discussion

According to the NRC (1994), the optimal requirement of Met for broilers is 0.5% from week 0 to 3, and 0.38% from week 3 to 6. In the modern poultry industry, the content of Met in the diet is 0.28% to 1.5%, which is at levels below the NRC (1994) recommendation of 0.5% (for example, 0.4, 0.3, and 0.2%). A significant decrease in feed intake was observed at only 0.3 and 0.2% Met; the latter was the most severe [1] and has a great negative influence on the broilers in many ways. In the poultry industry, Met is the first limiting essential amino acid, and must be explicitly added to the diets of broilers. Otherwise, many factors will be affected by the Met deficiency, such as the level of the crude protein, the content of the cysteine, the source of the Met, the level of selenium, and so on.

The bursa of Fabricius is the primary lymphoid organ in avian species. The bursa of Fabricius of birds has an essential role as a central lymphoid organ for the differentiation of B lymphocytes [23]. In addition, the bursa harbors immunecompetent B-lymphocytes which are capable of local antibody production [24]. The structure of the bursae of Fabricius is related to normal humoral immune function in chickens. Therefore, bursae may be used as a good model for studies on the effects of many factors of B-cell function. In the present research, the relative weight of bursae in Met-deficient chickens was significantly decreased. Histopathologically, lesions became serious from 21 days of age until the end of the experiment. Moreover, the swelled or vacuolated mitochondria of lymphocytes were ultrastructurally observed in Met deficiency. The lymphopenia could be a reason for the decreased weight of the bursae. The mitochondrial injury of the lymphocytes reflected the injury of the bursal cells. It was suggested that the humoral immune function was ultimately impaired by Met deficiency.

The eukaryotic cell cycle is divided into four major phases as follows: the G_1_ phase before DNA replication, the periods of DNA synthesis (S phase), the G_2_ phase before cell division, and cell division (M phase) [25]. In the present study, Met deficiency caused an increase in G_1_ phase cells that corresponded to a decrease in S phase cells and PI in the bursa of Fabricius. The results showed that the progression of lymphocytes from the G_0_/G_1_ to S phases was seriously impaired and the lymphocyte proliferation was restrained by Met deficiency, which could result in the reduced growth index and decreased number of lymphocytes of the bursa. The mechanism of effect of Met on lymphocyte proliferation is unclear. A possibility is that Met could affect lymphocyte proliferation by changing the production of proteins responsible for proliferation, such as cyclins, because Met could suppress protein synthesis [8].

Programmed cell death, or apoptosis, is a highly regulated process used to eliminate dysplastic or damaged cells from multicellular organisms [26]. In the present study, AnnexinV-FITC staining assay showed that the percentage of apoptotic cells in the bursa was significantly increased with Met deficiency. The mechanisms of apoptosis are associated with accumulation of the ceramide and activation of caspase [27]. It has been reported that Met deficiency could activate one or more pathways for initiating apoptosis that ultimately converge on a common execution pathway [17]. According to the results in the present study, we presumed that apoptotic pathway in the bursa mainly involved DNA damage-induced death signal, an initiator cysteine protease (caspase), a mitochondrial-derived signal, or involved the accumulation of cells in G_0_G_1_ phage.

## 5. Conclusions

According to the results in the present study and the aforementioned discussion, it was concluded that Met deficiency restrained the development of the bursa mainly by cell cycle arrest and caused increased apoptosis. The bursa’s function may ultimately be impaired and the immune function may be significantly impacted.
